# Online parrot trade as a source of psittacosis caused by a novel *Chlamydia psittaci* ST388

**DOI:** 10.3389/fmicb.2026.1777495

**Published:** 2026-03-03

**Authors:** Wenwu Yao, Zhifang Kong, Guoxiang Shi, Kai Song, Lingbo Wang, Zhuoying Wu, Qijie Zhao, Zhouwei Chen, Beibei Wu, Yajun Song

**Affiliations:** 1 State Key Laboratory of Pathogen and Biosecurity, AMMS, Beijing, China; 2 Department of Microbiology, Zhejiang Provincial Center for Disease Control and Prevention, Hangzhou, China; 3 Ninghai County Center for Disease Control and Prevention, Zhejiang, China; 4 Department of Infectious Diseases and Hepatology, First Hospital of Ninghai County, Zhejiang, China; 5 Hangzhou Digital-Micro Biotech Co., Ltd., Zhejiang, China

**Keywords:** *Chlamydia psittaci*, e-commerce, probe-based capture sequencing, psittacosis, ST388

## Abstract

**Introduction:**

The direct zoonotic risks associated with the growing online trade of live pets remain in adequately understood.

**Methods:**

We investigate a human case of severe psittacotic pneumonia in Zhejiang Province, China, in which the patient’s only avian exposure was through parrots recently purchased online. Using targeted probe-capture sequencing—a method designed for uncultivable, low-biomass pathogens—we recovered 13 high-quality *Chlamydia psittaci* genomes directly from the patient, the asymptomatic parrots, and their shared home environment.

**Results:**

Comparative genomic analysis revealed >99.99% average nucleotide identity across all samples, providing definitive evidence of parrot-to-human transmission. The causative strain was identified as a novel sequence type (ST388) within the known virulent genotype A.

**Conclusion:**

This investigation provides the first whole-genome confirmation of psittacosis transmission via online pet commerce. It establishes a practical genomic framework for investigating similar sporadic zoonoses and underscores the urgent need for targeted surveillance of this emerging and risk communication in this growing digital marketplace.

## 1 Introduction‌

Online commerce has revolutionized the sale of goods, including live animals, offering convenience but also creating potential new pathways for zoonotic disease emergence that challenge traditional surveillance systems (Wei et al., 2014).

*Chlamydia psittaci*, an obligate intracellular bacterium, is a prime example of a pathogen that can exploit such modern trade routes. It causes psittacosis, a globally distributed zoonosis affecting birds, mammals, and humans (Harkinezhad et al., 2009; Anstey et al., 2021; Stokes et al., 2021). Human psittacosis cases exhibit markedly heterogeneous clinical manifestations, ranging from mild flu-like symptoms to severe pneumonia, endocarditis, hepatitis, and fatal outcomes (Branley et al., 2016; Yao et al., 2022). Based on polymorphisms within the major outer membrane protein genes (*ompA*), *C. psittaci* strains are classified into nine major genotypes, among which genotypes A and E are most frequently associated with human infections (Van Lent et al., 2012; Wolff et al., 2015). *C. psittaci* exhibits broad host range, with confirmed infections across more than 400 different bird species including parrots, pigeons, ducks, turkeys, chickens, etc. (Knittler and Sachse, 2015; Borel et al., 2018).

In China, human psittacosis cases have been increasingly documented, particularly in regions like Zhejiang Province, where the subtropical monsoon climate supports diverse avian populations (Yao et al., 2022; Yao et al., 2023; Qin et al., 2024). This trend parallels a global rise in reported cases, partly attributable to the expanded clinical use of diagnostic tools like metagenomic next-generation sequencing (mNGS; Gu et al., 2019; Chen et al., 2020; Gu et al., 2020). However, despite advances in detection, a critical bottleneck persists: the acquisition of high-quality *C. psittaci* genomes for definitive molecular epidemiology remains exceptionally difficult due to the pathogen’s fastidious growth requirements and low bacterial loads in clinical specimens. Consequently, complete genomes from China are scarce, hindering a comprehensive understanding of its local genetic diversity, evolution, and precise transmission dynamics—especially for sporadic cases without clear exposure histories.

Current diagnostic methodologies, including quantitative PCR (qPCR), mNGS, and serology, are crucial for clinical intervention but often fall short of providing the high-resolution genomic data needed for outbreak source attribution (ELISA; Jelocnik et al., 2021).

While the proactive surveillance in China has focused on avian reservoirs, many human cases are identified incidentally, leaving their transmission chains incomplete or inferred (Gu et al., 2019; Chen et al., 2020; Gu et al., 2020). This gap between case identification and genomic confirmation is particularly problematic for assessing risks from novel exposure routes, such as online bird trade. Therefore, there is an urgent need for culture-independent methods capable of generating robust genomic data directly from complex samples to illuminate these hidden transmission networks.

To address this critical gap in genomic evidence for novel transmission routes, we applied a targeted probe-capture sequencing approach designed specifically for enriching low-abundance pathogen DNA. We investigated a human psittacosis case epidemiologically linked to online-purchased parrots in Zhejiang province. In this study, we reported 13 genomes of a novel sequence type ST388 of *C. psittaci* from a human psittacosis case, household members of cases, environmental and animal samples in Zhejiang province, by using oligonucleotide probe-based capture sequencing. Genomic analysis revealed that the patient was infected by her online-purchased parrots. This work provides the first whole-genome-level investigation of a *C. psittaci* infection chain originating from e-commerce, aiming not only to confirm the suspected zoonotic link but also to establish a replicable genomic framework for investigating similar e-commerce-associated zoonoses.

## 2 Materials and methods

### 2.1 Case description

The patient is a 44-year-old female who lives with her families (her husband and two sons) in Yuelong Subdistrict, Ninghai County, Zhejiang Province. The family operates a small variety store, where they keep four parrots purchased online. The patient both works and resides within the store. On July 20, 2024, she developed chills and a fever, with her body temperature reaching a maximum of 39.5 °C (103.1 °F). She sought medical attention at a local hospital on July 24, where she was diagnosed with “infectious fever.” The following day, on July 25, she was transferred to the Infectious Diseases Department of Ninghai County First Hospital for continuous monitoring of her vital signs.

### 2.2 Sample collection and qPCR

A total of 20 clinical and environmental samples were collected, including one bronchoalveolar lavage sample from the patient, three throat swabs from her husband and two children, paired oropharyngeal/cloacal swabs from four parrots, and eight environmental swabs (Table 1). DNA was extracted using the QIAamp DNA Mini Kit (QIAGEN, Germany) following manufacturer’s protocol in Zhejiang Provincial Center for Disease Control and Prevention (CDC), followed by qPCR targeting the 16S rRNA of *C. psittaci* according to previously study (Yao et al., 2022).

**Table 1:** Statistical table for the results of nucleic acid testing and probe-based capture sequencing.

Sample sources	Sample type	qPCR	CT value	Genome ID	Accession number
Patient	bronchoalveolar lavage	positive	30	NH-1	NMDC60210010
Children 1	throat swab	Negative	-	-	-
Spouse	throat swab	Negative	-	-	-
Children 2	throat swab	Negative	-	-	-
Purple parrot	throat swab	positive	34	-	-
Purple parrot	Anal swab	positive	32	NH-2	NMDC60210011
Green parrot	throat swab	positive	28	NH-3	NMDC60210012
Green parrot	Anal swab	positive	30	NH-4	NMDC60210013
Blue parrot	throat swab	positive	30	NH-5	NMDC60210014
Blue parrot	Anal swab	positive	30	NH-6	NMDC60210015
Birdcage 1	smear	positive	32	NH-7	NMDC60210016
Foods	smear	positive	34	-	-
Grinking water	smear	Negative	-	-	-
Feces	smear	positive	31	NH-8	NMDC60210017
Ground	smear	positive	31	NH-9	NMDC60210018
Cardboard box	smear	positive	32	NH-10	NMDC60210019
Birdcage 2	smear	positive	34	NH-11	NMDC60210020
Desktop	smear	positive	30	NH-12	NMDC60210021
Yellow parrot	throat swab	positive	36	-	-
Yellow parrot	Anal swab	positive	31	NH-13	NMDC60210022

Yellow indicates Chlamydia psittaci nucleic acid-positive samples, while orange represents samples from which genomes were obtained through sequencing.

### 2.3 Probe-based capture sequencing and assembly

For whole-genome sequencing, custom RNA probes were designed by Agilent Technologies (Beijing, China) using the Tier2 design (0.5–2.99 Mbp). Twenty-five complete reference genomes of *C. psittaci* were selected to ensure comprehensive chromosomal/plasmid coverage and genomic diversity. 16 nucleic acid-positive *C. psittaci* samples underwent RNA probe capture-based whole-genome enrichment, followed by quality assessment using Qubit and *q*PCR. Subsequent next-generation genome sequencing was performed on the Illumina platform. Raw data quality was assessed with CheckM, and species confirmation performed via fidBac.^1^ The generated sequencing data is first assembled *de novo* without a reference genome used SPAdes v3.13.1. After the assembly is completed, a blastn analysis is performed to identify the most closely related reference genome, then the most similar genome is used as the reference for reference-based assembly by BWA (BWA-MEM).

### 2.4 Genomic analysis

Genomic annotation utilized Prokka v1.14.6 for gene prediction, with functional characterization through eggNOG v5.0.2 (COG/GO terms), Diamond (KEGG pathways), RGI v6.0.3 and AMRFinder v3.12 (antimicrobial resistance/virulence factors). Core-pan genome analysis was conducted with Roary v3.13.0, and maximum-likelihood phylogenies reconstructed from core genes using FastTree v2.2.0. For *ompA* gene analysis, structural domains were predicted via MEME Suite.^2^ Neighbor-joining phylogenies incorporated sequences from all 103 isolates aligned by BLAST against NCBI references. Final trees integrated MLST data and ompA domains using iTOL.^3^

### 2.5 Multilocus sequence typing

Multilocus sequence typing (MLST) employed FastMLST v0.0.1 and PubMLST^4^ across 103 strains (90 public genomes + 13 novel isolates). The 7 housekeeping genes were amplified by PCR and sequenced. The obtained housekeeping gene sequences were submitted to the PubMLST website for MLST typing analysis. Based on the number of allele profiles obtained for the housekeeping genes, the website assigned a new MLST number.

## 3 Results

### 3.1 Clinical features

**Figure 1:**
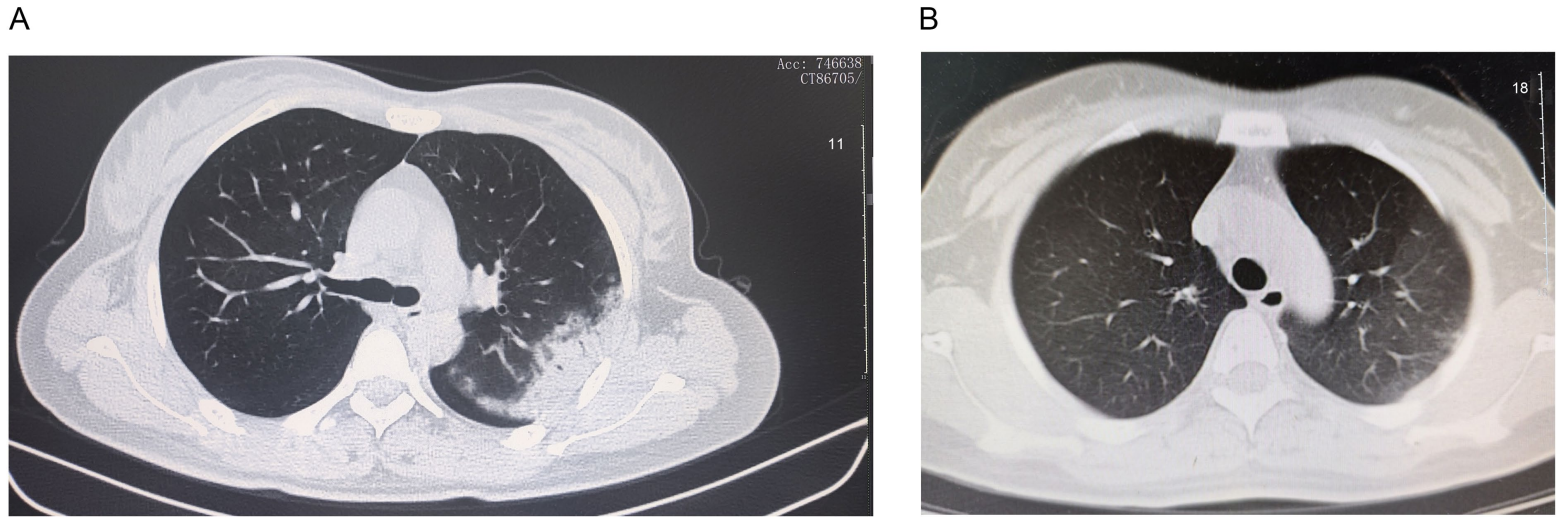
Clinical and radiological findings in the index patient with psittacosis. **(A)** Chest CT obtained on day 4 after symptom onset (July 24, 2024), showing extensive consolidation with surrounding ground-glass opacity in the left upper lobe. **(B)** Follow-up CT on day 11 (August 1, 2024) after initiation of doxycycline therapy, demonstrating significant resolution of the consolidation.

A 44-year-old female, residing and working in a family-run store, presented on July 24, 2024, with a history of high-grade fever (up to 39.5 °C) and chills of 4 days’ duration. Initial laboratory investigations were consistent with a significant systemic inflammatory response: leukocyte count 7.06 × 10⁹/L, with neutrophilia (5.18 × 10⁹/L, 73.4%), lymphopenia (1.06 × 10⁹/L, 15.0%), and a markedly elevated C-reactive protein level of 63.7 mg/L. Admission chest CT revealed extensive, inhomogeneous consolidation with ground-glass opacity halo in the left upper lobe, indicative of severe community-acquired pneumonia (Figure 1A). Empirical therapy with moxifloxacin was initiated. The definitive diagnosis was achieved 3 days post-admission, when metagenomic next-generation sequencing (mNGS) of bronchoalveolar lavage fluid identified 412 sequence reads uniquely mapping to *C. psittaci*, with a genomic coverage of 1.2%. Antimicrobial therapy was immediately escalated to doxycycline (100 mg twice daily). A follow-up CT scan on day 11 of illness demonstrated dramatic resolution of the consolidation, with only minor residual ground-glass opacities (Figure 1B). The patient made an uneventful recovery and was discharged on August 3.

### 3.2 Epidemiological investigation

**Figure 2:**
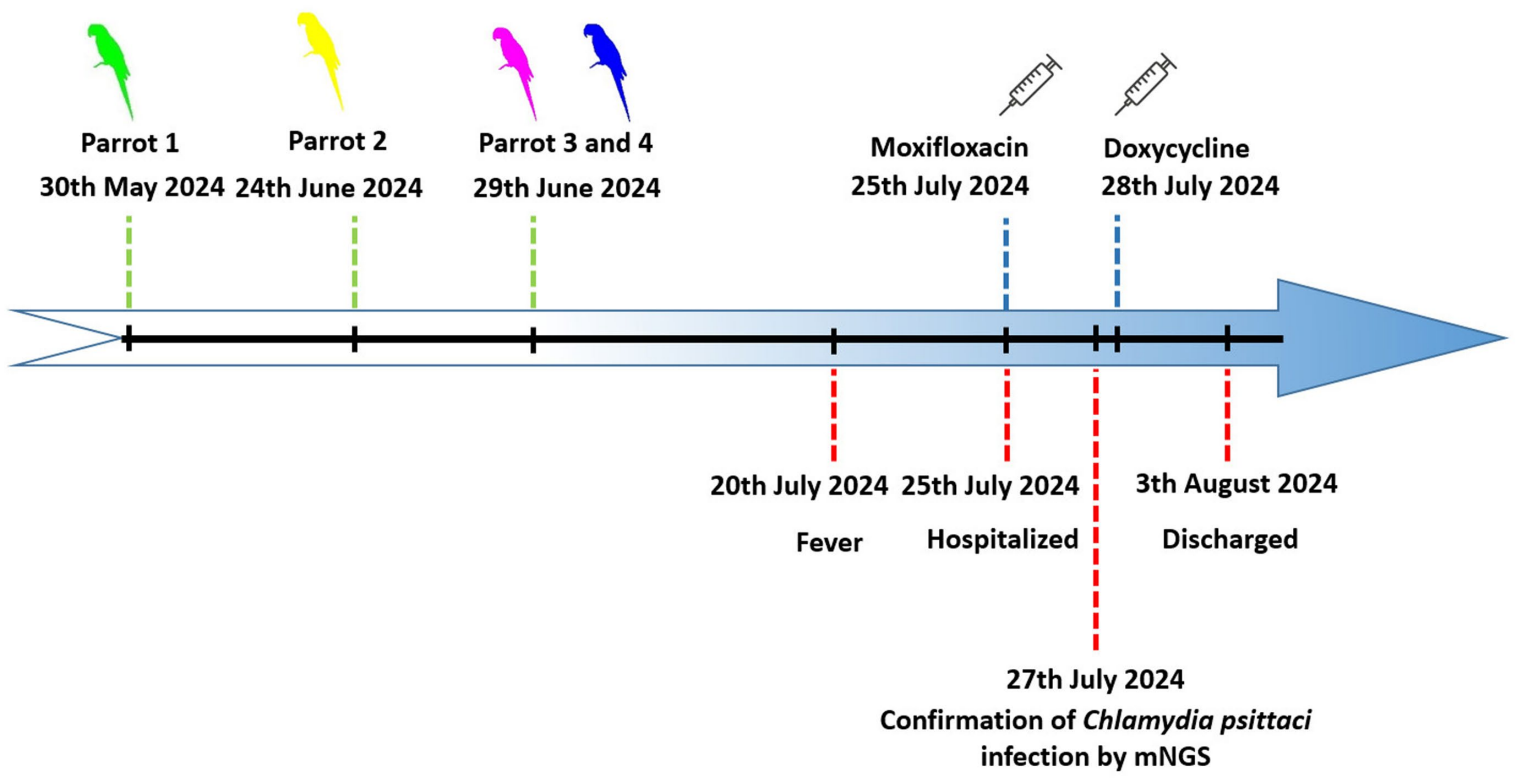
Epidemiologic timeline linking online parrot purchases to human illness. The timeline illustrates the dates of online parrot acquisitions (May 30, June 24, and June 29, 2024), symptom onset in the patient (July 20, 2024), key clinical interventions (hospital admission, mNGS diagnosis, antibiotic change), and sample collection. The interval from the final purchase to symptom onset was 21 days.

Epidemiological investigation established that the patient acquired multiple parrots via online commerce: a green peony parrot from Zhengzhou (Henan Province) on May 30; a yellow parrot on June 24; and purple/blue parrots on June 29 from Suzhou (Anhui Province; Figure 2). All birds were co-housed in a single cage and exclusively managed by the patient, with symptom onset occurring 20 days post-final acquisition. All four parrots have remained healthy since purchase and have not exhibited any symptoms such as fever, coughing, or diarrhea. The patient independently handled all daily tasks related to feeding the parrots and cleaning their living area. The patient reported not using any personal protective measures, such as wearing a mask or gloves, during these activities. Aside from the parrots, there were no other domestic animals, poultry, or birds kept in the household. Additionally, no raw live poultry or livestock were sold in the local supermarket. The patient denied any travel history in the 6 months prior to onset of illness, and reported no contact with individuals exhibiting similar symptoms in the past month. Subsequent analysis of the 20 collected clinical and environmental samples determined the presence of *C. psittaci* nucleic acid in 16 samples by quantitative PCR (qPCR; Table 1).

### 3.3 Genome characterization

**Figure 3:**
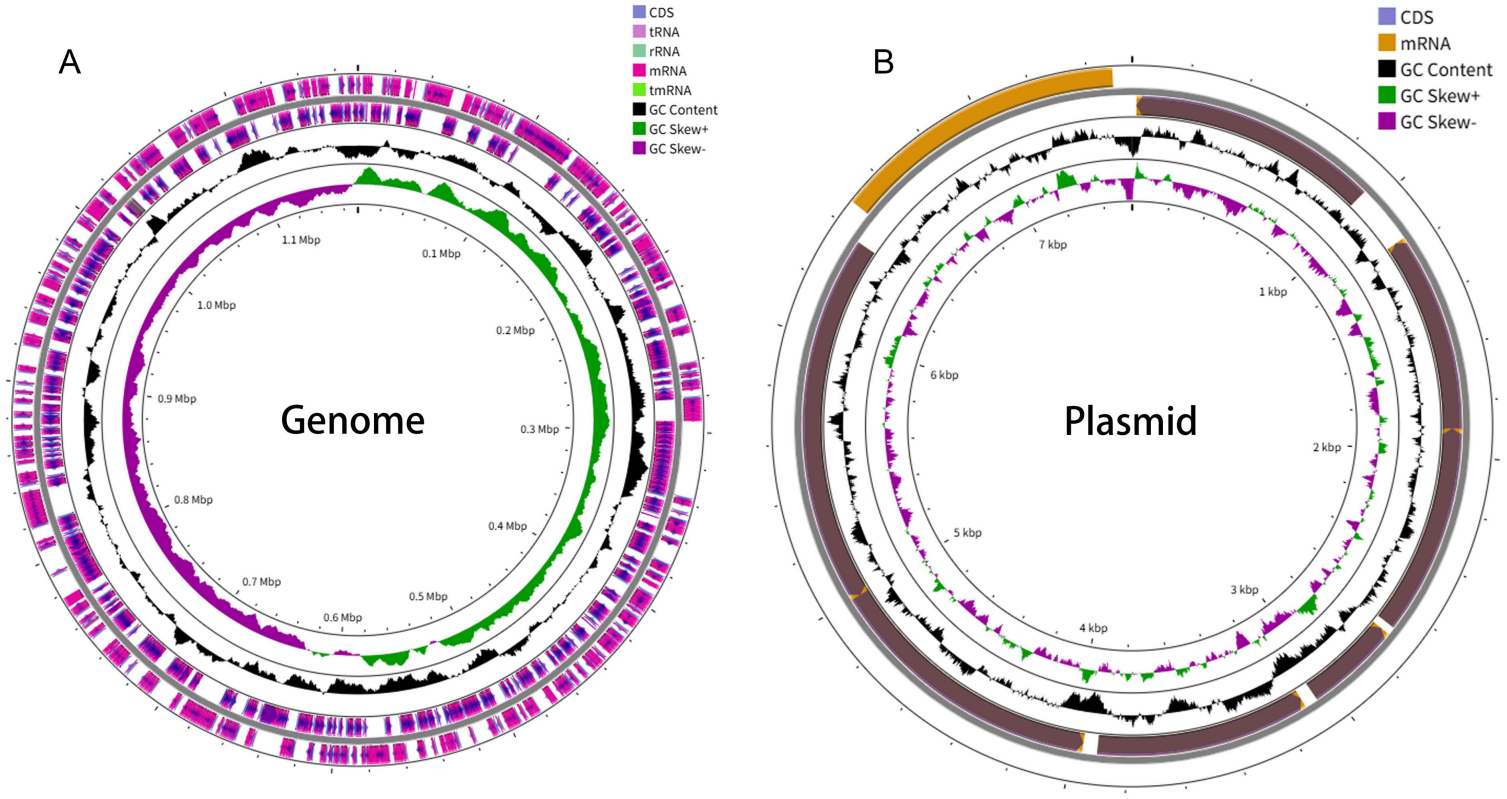
Genomic features of *Chlamydia psittaci* strain NH-1 from the index patient. **(A)** Circular map of the 1,172,199 bp chromosome, showing predicted coding sequences (CDS, blue), tRNAs (red), and GC content (inner ring). **(B)** Map of the conserved 7.1 kb plasmid. Genome sketches were generated using Proksee (https://proksee.ca/).

**Figure 4:**
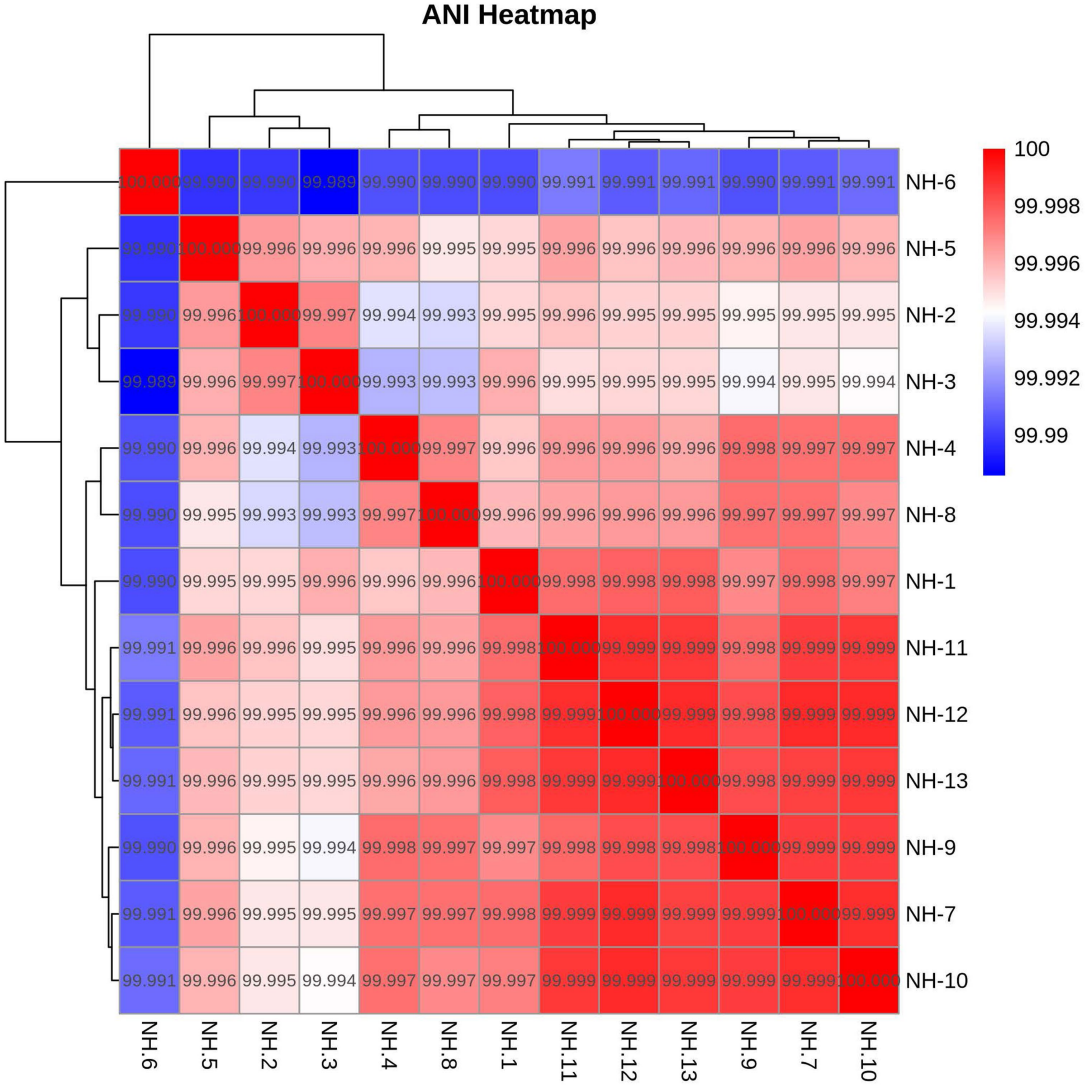
Pairwise average nucleotide identity (ANI) matrix for the 13 *C. psittaci* genomes from this outbreak. Heatmap generated using fastANI v1.32, demonstrating >99.99% ANI among all human, avian, and environmental isolates, confirming a single infection source.

Given the fastidious nature of *C. psittaci*, we employed a targeted enrichment strategy. Custom-designed RNA probes were used to capture genomic material directly from the 16 qPCR-positive samples, bypassing the need for culture. This approach successfully generated 13 complete genomes (assembly sizes: 1,166,062–1,172,392 bp) from samples with a qPCR Ct value ≤ 34, achieving an average sequencing depth of 150 × and coverage of >95% of the reference chromosome. The genome from the index patient (NH-1) was 1,172,199 bp in length and contained 992 predicted coding sequences and 38 tRNAs (Figure 3A). All 13 genomes carried a conserved 7.1 kb plasmid typical of *C. psittaci* (Figure 3B). Pairwise average nucleotide identity (ANI) among all isolates was >99.99% (Figure 4), suggesting these genomes derived from the same species. Multilocus sequence typing (MLST) identified a novel sequence type, ST388, which is a single-locus variant (SLV) of the globally disseminated ST24 lineage—a lineage implicated in infections across birds, livestock, and humans (White et al., 2023). This finding suggests a recent microevolution within this successfully and broadly host-adapted lineage. Concurrent analysis of the *ompA* gene classified the genomes as genotype A.

### 3.4 Phylogenetic analysis

**Figure 5:**
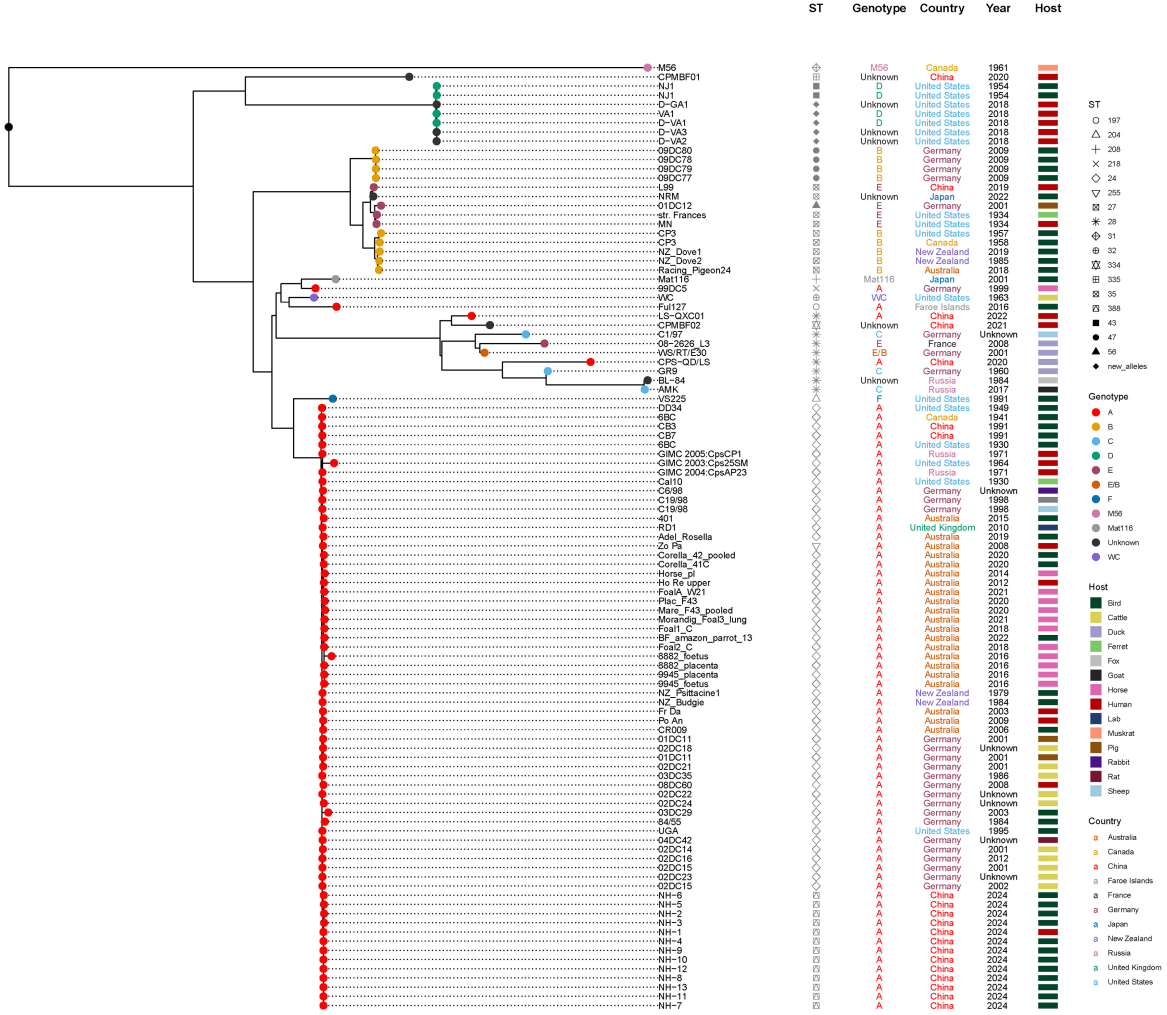
Core-genome maximum-likelihood phylogeny of 103 global *C. psittaci* isolates. The tree includes the 13 novel ST388 genomes from this study (highlighted in red) and 90 publicly available genomes. Tip points are colored and shaped according to multi-locus sequence type (MLST), host, and country of origin, as indicated in the legend. The scale bar represents nucleotide substitutions per site. A detailed ST24/ST388 clade subtree is provided in Figure 6.

**Figure 6:**
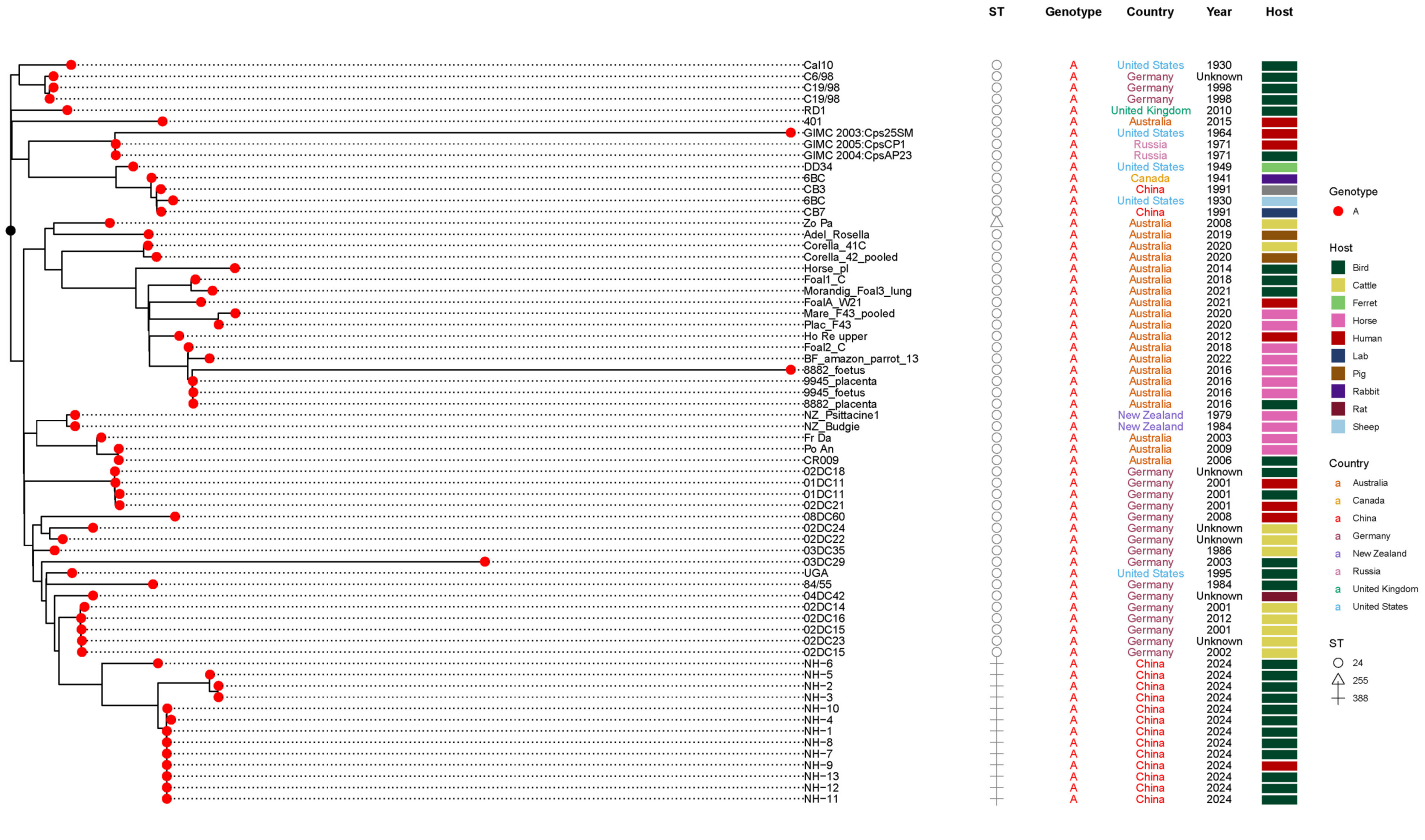
Detailed phylogeny of the *C. psittaci* ST24 complex, including the novel ST388. Unrooted maximum-likelihood tree focusing on 67 genomes belonging to the ST24 lineage and its single-locus variants (SLVs). The novel ST388 strains from this study (red) cluster closely with ST24 strains previously isolated from cattle in Germany, indicating microevolution within this globally disseminated lineage.

To contextualize our isolates, we constructed a core-genome (regions present in >99% of isolates) maximum-likelihood (ML) phylogeny incorporating the 13 ST388 genomes and 90 publicly available *C. psittaci* genomes spanning diverse hosts, geographical regions, and collection years (1930–2022). All the 13 *C. psittaci* genomes identified in this study were clustered into same cluster, along with 54 ST24 (including one SLV ST255) genomes obtained from different hosts (Figure 5). As shown in Figure 4, ST24 and its SLVs are dominant in the population (67/113). We further constructed an unrooted ML tree of the 67 ST24 complex genomes (Figure 6). ST24 strain was firstly isolated from a ferret of United States. It was then found in Germany, Russia, United Kingdoms, Australia and New Zealand, from different host, including humans, birds, ferrets, cattle, rats, horses, and rabbits, etc. Although two ST24 stains had been found in 1991, the genomes identified in this study is distinct from these two strains (Jelocnik et al., 2017). Notably, the phylogenetic position of the novel ST388 strains shows closest proximity to ST24 strains previously isolated from cattle in Germany. This clustering pattern, bridging a novel lineage from Chinese parrots and a human case with established European bovine strains, highlights the extensive geographic dissemination and multi-host adaptability of the ST24 lineage. It raises the possibility of undetected international transmission routes or convergent evolution across continents, potentially facilitated by global animal trade or environmental persistence of this successful clade.

## 4 Discussion

*Chlamydia psittaci* is an important zoonotic pathogen posing significant global public health threats, as evidenced by increasing cases in China, USA, Europe, and Australia (Yung and Grayson, 1988; Branley et al., 2014; Shaw et al., 2019). The observed rise in Chinese cases partially reflects expanded clinical utilization of mNGS (Gu et al., 2019; Chen et al., 2020). However, research on *C. psittaci* in China remains limited, hindered by the pathogen’s fastidious growth requirements and its exclusion from China’s national notifiable infectious disease list (Liu et al., 2023). Zhejiang Province, with its humid subtropical monsoon climate conducive to diverse avian populations, has reported several human psittacosis cases linked to bird exposure in recent years (Yao et al., 2022; Yao et al., 2023). Despite this, whole-genome studies of *C. psittaci*, particularly in China, are still rare, primarily due to the challenges of isolating the bacterium from clinical samples where it often presents in low bacteria loads.

To address this critical gap, we employed a culture-independent, probe-capture sequencing approach, enabling the direct recovery of high-quality genomes from low-biomass samples. This method successfully generated 13 complete *C. psittaci* genomes from clinical, avian, and environmental specimens with Ct values ≤ 34, overcoming a key diagnostic bottleneck by obtaining the full genome of *Chlamydia psittaci* without culturing it, using a targeted probe capture sequencing method. Our work aligns with and extends recent international efforts using similar culture-independent genomics for *C. psittaci* (White et al., 2022; Kasimov et al., 2023). To our knowledge, this study provides the first whole-genome evidence of direct *C. psittaci* transmission from parrots to a human. While prior studies have inferred zoonotic links through epidemiology or lower-resolution genotyping (e.g., MLST/ompA) [1–3], we resolved a complete transmission chain with genomic certainty from pairing clinical, avian, and environmental samples.

Our study reports the first documented case of human *C. psittaci* infection (parrot fever) linked to an online parrot purchase. This genomic evidence unequivocally establishes parrot-to-human transmission, demonstrated by >99.99% average nucleotide identity across all 13 genomes from the patient, parrots and contaminated environments. Precise source attribution proved challenging, as the patient acquired four parrots through three separate online transactions. The incubation period of psittacosis is normally 5–14 days, while the longest empirically recorded incubation period is 22 days, documented during an outbreak associated with a bird fair in France through a retrospective cohort study (Belchior et al., 2011; Knittler and Sachse, 2015). We therefore propose the parrots purchased on June 29 (the purple and the blue) as the infection source, given the 21-day interval to the patient’s symptom onset (July 20) falls within the maximum documented incubation period.

Critically, we observed no secondary transmission despite significant exposure opportunities, as the patient’s cohabiting family members (husband and two sons) showed no evidence of infection despite shared avian contact and 5 days of household exposure to the symptomatic patient prior to hospitalization. While limited reports suggest possible human-to-human transmission under specific circumstances, Zhang et al. reported the first documented report of human-to-human transmission of *C. psittaci* in China (Zhang et al., 2022). Our findings, demonstrating no secondary transmission despite significant household exposure, support the view that efficient human-to-human transmission of *C. psittaci* is uncommon. Consequently, public health priorities should emphasize awareness campaigns for avicultural workers, mandatory personal protective equipment during waste handling, and enhanced regulation of online live bird markets.

The 13 genomes were identified as a novel sequence type, ST388, identified as a single-locus variant (SLV) of the globally disseminated ST24 lineage, and classified as genotype A. Genotype A is a classic, virulent strain closely associated with avian-to-human zoonotic transmission (Kasimov et al., 2023; Hou et al., 2024). The ST24 lineage has been isolated worldwide from a diverse range of hosts, including humans, birds, and livestock (White et al., 2023). The emergence of ST388 within this lineage signifies ongoing microevolution. Its close phylogenetic proximity to ST24 strains from cattle in Germany, as revealed in our analysis, highlights the extensive geographic dissemination and multi-host adaptability of the ST24 complex. This pattern suggests potential undetected convergent evolution or international transmission routes, possibly facilitated by global animal trade. Understanding the mechanisms behind the successful adaptation of ST24 and its variants (like ST388) across different hosts is of vital scientific and public health importance.

Our findings document the first human psittacosis case linked to online parrot commerce, thereby defining an emerging and diffuse risk pathway that challenges traditional surveillance focused on physical markets and known avian reservoirs. Consequently, public health strategies must evolve. We recommend: (1) strengthening awareness and mandating personal protective equipment for individuals involved in bird care; (2) enhancing the regulation of online live animal markets, including exploring mechanisms for health certification; and (3) considering the inclusion of psittacosis in notifiable disease surveillance systems in key regions to improve detection and response.

## 5 Conclusion

In summary, this study investigated a psittacosis case in Ninghai, Zhejiang Province, by recovering 13 *C. psittaci* genomes via a culture-independent probe-capture sequencing approach. Genomic and epidemiological analyses confirmed the infection was caused by a novel strain (ST388, genotype A) transmitted from online-purchased parrots. This represents the first whole-genome-confirmed case of psittacosis transmission via this modern trade route. Our work demonstrates the power of culture-independent genomics for outbreak investigation and underscores the urgent need to address the growing risk of zoonoses, including psittacosis, in the era of digital commerce.

## Data Availability

The data presented in the study are deposited in the National Microbiology Data Center repository, accession number(s) NMDC60210010-NMDC60210022.

## Footnotes

1
http://fbac.dmicrobe.cn/

2
https://meme-suite.org/

3
https://itol.embl.de/

4
https://pubmlst.org/
